# Exploring change over time in community mobilization domains: results from a maternity waiting home intervention in rural Zambia

**DOI:** 10.1186/s12939-021-01557-5

**Published:** 2021-10-19

**Authors:** Dana Beck, Philip T. Veliz, Michelle Munro-Kramer, Carol Boyd, Isaac Sakala, Nchimunya Chiboola, Jody Lori

**Affiliations:** 1 Ann Arbor, USA; 2 Africare/Zambia, Flat A, Plot 2407/10 MBX, Off Twin Palm Road, Ibex Hill, Box 33291, Lusaka, Zambia

**Keywords:** Community mobilization, Maternal health, Social change

## Abstract

**Background:**

Community mobilization (CM) is recommended as a best practice intervention for low resource settings to reduce maternal mortality. Measurement of process outcomes are lacking and little is known about how CM impacts individuals or how community members perceive its function. Given the complex and recursive nature of CM interventions, research that describes the CM process at multiple levels is needed. This study examines change in CM domains at baseline and endline in rural Zambia.

**Methods:**

This secondary analysis uses data from a large maternity waiting homes intervention in rural Zambia that employed CM over 3 years as part of a package of interventions. A 19-item CM survey was collected from three groups (women with babies < 1, health workers, community members; *n* = 1202) with focus groups (*n* = 76) at two timepoints from ten intervention and ten comparison sites. Factor analysis refined factors used to assess temporal change through multivariable regression. Independent covariates included time (baseline vs endline), intervention vs comparison site, group (women with babies, healthworkers, community members), and demographic variables. Interaction effects were checked for time and group for each factor.

**Results:**

Final analyses included 1202 individuals from two districts in Zambia. Factor analysis maintained domains of governance, collective efficacy, self-efficacy, and power in relationships. CM domains of self-efficacy, power in relationships, and governance showed significant change over time in multivariable models. All increases in the self-efficacy factor were isolated within intervention communities (b = 0.34, *p* < 0.001) at endline. Between groups comparison showed the women with babies groups consistently had lower factor scores than the healthworkers or community member groups.

**Conclusions:**

Community mobilization interventions increase participation in communities to address health as a human right as called for in the 1978 Alma Ata Declaration. Grounded in empowerment, CM addresses socially prescribed power imbalances and health equity through a capacity building approach. These data reflect CM interventions function and have impact in different ways for different groups within the same community. Engaging directly with marginalized groups, using the community action cycle, and simultaneous quality improvement at the facility level may increase benefit for all groups, yet requires further testing in rural Zambia.

## Background

Community mobilization (CM) is recommended by the World Health Organization as a best practice intervention for low resource settings to reduce maternal and neonatal mortality [1] . Tailored to local context, the CM process is structured around the community action cycle (CAC) [1], where local communities identify, prioritize, and act to solve health and social problems in collaboration with external facilitators. Globally, in communities where women’s groups have been facilitated using the CAC, they become the locus of power in identifying problems, mobilizing resources, and acting to solve them effectively [2–4]. Programmatic outputs from women’s groups using the CAC include the distribution of home clean delivery kits [5], the creation of emergency transport funds [6, 7] or implementation of dimba gardens to grow iron rich food for prevention of post-partum hemorrhage [7] A meta-analysis of seven randomized controlled trials representing 119,428 births in rural settings in Malawi, Bangladesh, India, and Nepal found CM interventions using the CAC via women’s groups indicated a 20% reduction in neonatal mortality, also finding that for studies reporting high participation (minimum 30%), maternal death was reduced [6]. In India, CM using women’s groups have been scaled up with support from the Ministry of Health, where results also show increased confidence in navigating the health system and improved quality of care [8].

Despite promising results from CM interventions for maternal and child health, unstandardized reporting of results has hindered their expansion [9, 10] . However, use of linking constructs is shown to clarify process outcomes in CM interventions [9] . Linking constructs characterize a causal pathway to illustrate how a process works [9]. A review of CM interventions for sexual, reproductive, and maternal health found 11 studies encompassing 23 linking constructs in their evaluation, ranging from single to multi-item scales, or independent items [9]. The linking constructs isolated were organized within 8 domains (collective action, collective agency, collective efficacy, collective identity, governance, perceived similarity, social acceptance/cohesion, social networks/support) said to influence CM outcomes. A validated scale evaluating CM domains pertinent to the rural South African context includes domains of shared concern, critical consciousness, leadership, organizations/networks, collective action, social cohesion, and social control [11]. Despite these available tools, CM interventions rarely incorporate process evaluation using linking constructs representing CM domains.

Use of CM interventions to address maternal mortality in sub-Saharan Africa remain limited in part due to concerns related to a lack of understanding of how the process functions. Yet, countries in sub-Saharan Africa bear 66% of the global burden of maternal deaths and expansion of strategies that report on CM as a process are needed in order to expand the evidence base for this lifesaving intervention. In particular, rural Zambian women and girls continue to face challenges in experiencing safe, facility supported pregnancy and childbirth, with Zambia’s maternal mortality ratio at 252 deaths per 100,000 live births and the infant mortality rate at 42.4 per 1000 live births [12].

**Fig. 1:**
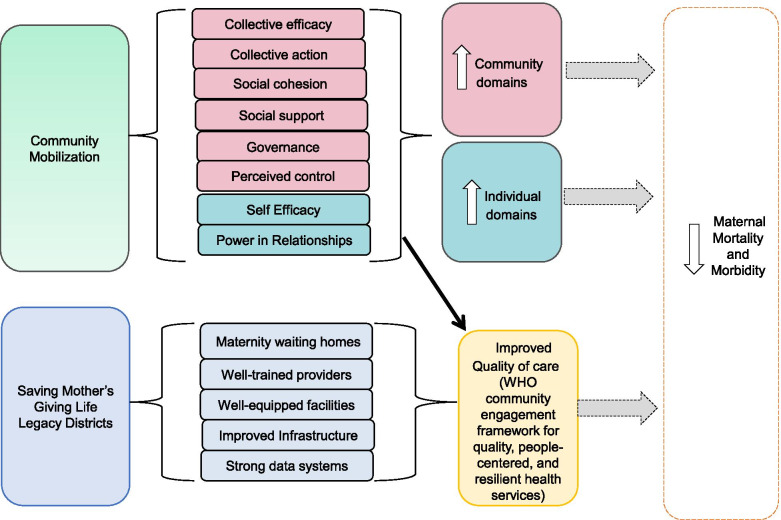
Community Mobilization Theory of Change

The following study addresses current gaps in the science of CM measurement and reporting through examination of CM domain data collected as a part of a large, longitudinal Maternity Waiting Homes (MWH) intervention coupled with CM in two districts in rural Zambia. Guided by an investigator derived CM Theory of Change (Fig. 1), the aims of this study were twofold:1) Examine the factor structure (i.e., latent constructs) of a CM survey among rural Zambians using baseline and endline data and 2) Examine the change in domains of CM among a sample of rural Zambians from three groups (women with babies, community members, health workers) within 10 communities surrounding the ZaMS using baseline (*n* = 553) and endline CM survey data (*n* = 649).

## Methods

This secondary analysis uses cross-sectional data at two time points from a parent study investigating the impact of maternity waiting homes (MWHs) to examine proposed domains of CM and their change over 3 years among rural Zambians. This included 10 communities in Mansa and Lundazi districts with recently constructed MWHs, known as Zambian Mother’s Shelters (ZaMS). This study was deemed exempt and not regulated by the University of Michigan IRB (HUM00165339). The dataset for these analyses come from an evaluation of MWHs which began in May 2015 and concluded in July 2018 in partnership between researchers at the University of Michigan School of Nursing and Africare-Zambia. The parent study focused on interventions for strengthening the health system and increasing facility delivery for women living the farthest from care using a package of interventions, including CM. Using a two-group comparison design, the parent study evaluated maternal outcomes at 10 rural health facilities with ZaMS and 10 rural health facilities without ZaMS in the Mansa and Lundazi districts. Interventions at the ZaMS sites focused on strengthening referral networks from rural health facilities to district level facilities.

### Setting

Mansa and Lundazi districts were chosen due to their prior identification as Saving Mothers Giving Life (SGML) legacy districts, which included the training of Safe Motherhood Action Group members- community volunteers dedicated to reducing maternal mortality [13]. Ten sites in Mansa and ten sites in Lundazi were identified by the Ministry of Health and the parent study’s implementing partners Africare-Zambia, and jointly determined to be included based on predetermined site selection criteria (see Table 1). The five ZaMS (intervention) sites were matched with five comparison sites based on distance from comprehensive emergency obstetric and newborn care (CEmONC) in kilometers and time in minutes, delivery volume, number of women of reproductive age, and absence of a functional MWH [13].

**Table 1:** Intervention (ZaMS) and Comparison (non ZaMS) sites in Mansa, Zambia and Lundazi, Zambia (Scott et al., 2018)

Basic emergency obstetric and newborn care (BeMONC) sites with ZaMS	Population	Distance in Km (minutes from BeMONC)	BEmONC sites with no ZaMS (matched comparison sites)	Population	Distance in Km (Minutes from BeMONC)
***MANSA***					
Lubende	4192	44(40)	Musaila	2868	0.88(55)
Mano	6619	46(40)	Kundamfumu	6600	45(30)
Fimpulu	5597	32(25)	Kabunda	5075	15(15)
Mutiti	6776	60(45)	Mibenge	7722	58(50)
Lukola	5957	12(25)	M’wanguni	7722	12(15)
***LUNDAZI***					
Mwase Lundazi	19,578	31(25)	Kapichila	10,287	27(30)
Nkhanga	11,913	40(50)	Kamsaro	5701	24(40)
Nyangwe	6407	55(60)	Lukwizizi	4463	35(50)
Lusuntha	10,323	16(10)	Phikamalaza	6051	16(20)
Zumwanda	6670	30(30)	Chikomeni	7475	35(30)

* *BeMONC* Basic emergency obstetric and newborn care; Zambian Mothers Shelters = ZaMS

### Sampling

Participants were recruited via convenience sampling, wherein each village in the catchment area was notified by the local health facility and local leaders by word of mouth of the opportunity to participate in research and provided with the date and time to arrive at the health facility if they were interested and eligible to participate. Research participants were eligible if they were from one of three groups: 1) women with babies (< 1 years of age at baseline and < 2 years of age at endline), 2) community members, or 3) health workers. These three groups were selected in order to ascertain how the CM and MWH intervention impacted various groups within communities to provide information for future interventions.

### Inclusion and exclusion criteria

Inclusion criteria for each group:

1. Women with babies: The women with babies group included mothers over the age of 15 years old. Mothers under the age of 18 years old who become ‘parent-children’ in Zambia are considered emancipated under the law and do not require parental consent for inclusion in research [14].
2. Community members: Men or women who lived in the community for at least 1 year and were over the age of 18 years old.
3. Health workers: Health workers included Safe Motherhood Action Group members, community health workers, or environmental health technicians that were age 18 years old or older.

Exclusion criteria for all groups included people who were visiting the health center on the day of research activities and were ill requiring treatment.

### Data collection

Participants were given a brief description of the study and informed consent in the local language (Tumbuka, Bemba, or Nyanja) by the Africare-Zambia research staff. Interested individuals read through consent forms or had forms read to them in a private location where they were given the opportunity to ask questions. Africare-Zambia staff that spoke English and the local language conducted both the informed consent as well as assisted with the individual interview for illiterate participants. Those interested in participating signed the consent, or affirmed consent through use of a single finger print using an ink pad. Participants completed the individual survey on their own (unless illiterate, then were assisted by research assistant). Participants were provided with a snack and beverage as compensation for their time.

### CM activities

The CM process employed at the ZaMS sites focused on implementing strategies that would elevate demand generation strategies to increase use of ZaMS and their associated health facilities. Villages that were ten kilometers or greater from the health facilities/ZaMS were prioritized for sensitization and CM, led by Safe Motherhood Action Group members, who focused on the encouragement of pregnancy and birth registration, birth preparedness, and provided information about the ZaMS. As a part of the CM process, Safe Motherhood Action Group members received training from the Ministry of Health on the best way to conduct community meetings, which they held monthly in villages in their catchment and/or at the health facilities to discuss healthy pregnancy and birth. District Health offices provided support through meetings with traditional leaders, where headmen were encouraged to support the work of the Safe Motherhood Action Group members. Headmen met monthly to discuss progress in relation to facility birth and male involvement with reproductive health services in their communities. In addition, each ZaMS site formed governance committees that were democratically to include a treasurer, secretary, president, and vice president. These groups met monthly and implemented various income generating strategies to diversify their income generation and work toward sustainability of the ZaMS.

### Measures

The parent study contained pre-post (2015–2018) individual survey data systematically collected using a 19-item questionnaire (See Table 5 in Appendix). For the purposes of analysis, the 19-items used for this study was referred to as the CM survey. The CM survey used a dichotomous yes/no response for each item. The CM survey data were collected from three groups (women with babies, health workers, and community members) from intervention and comparison communities at baseline and endline of the parent intervention. Characteristics of the population sampled were collected as categorical (highest level of formal education; marital status; group - women with babies, community members, health workers) and continuous (age, years lived in the community, number of living children) variables. The variable concerning level of education was collapsed to include three categories (none, some/completed primary, some or completed secondary or tertiary), marital status was dichotomized to married/unmarried.

### Analysis

Exploratory factor analysis (EFA) was first conducted on the baseline sample of 553 and the endline sample of 649.To account for missing data, listwise deletion was used. All *p*-values were set at 0.05. We examined sampling adequacy using the Kaiser-Meyer-Olkin (KMO) and Bartlett test [15]. Baseline, endline, and combined sample (baseline and endline) samples were explored separately using oblique rotation to find the best factor solution. The extraction method was set to principal axis. Upon completion of the EFA, the number of factors to be retained was decided, the total number of possible factors represented by the total number of variables analyzed, and only theoretically meaningful factors retained [16]. Criteria for factor extraction included Bartlett’s test of sphericity, scree test, Kaiser-Guttman criteria, and item factor loading > 0.5. Structural validity was assessed by examining factor loading in relation to theoretical domains. We tested for internal consistency reliability using Cronbach’s alpha, with 0.70 set as an acceptable score. Retained factors were used to evaluate change over time in the baseline and endline CM survey data using a multivariable descriptive model.

Based on the results from the EFA, we tabulated the number of yes/no answers for each item on the revised (post factor analysis) survey to create a score for each factor. This was done for the baseline and endline surveys separately. This score was then used to compare means over time. There was a low amount of missingness on the CM survey ranging from one self-efficacy item (*n* = 5, 0.4%), to the highest amount of missing responses was from a power in relationships item (*n* = 44, 3.7%) (Table 5 in Appendix). Between group comparison was conducted to assess the impact of CM on the different groups (women with babies, health workers, community members) in relation to demographic information such as age, level of formal education, and years lived in the community. Categorical variables were analyzed using chi-square, to compare proportions. Continuous variables were analyzed to compare means using independent t-tests, with Mann Whitney U tests used in the case of skewed data. We then created four separate multivariable regression models for each factor, controlling for independent variables including: 1) pre- and post-intervention (time); 2) intervention vs comparison site; 3) group (women with babies, community members, health workers); and 4) demographic variables such as years lived in the community, age, marital status, number of living children, and level of formal education. Independent variables had missingness that ranged from *n* = 0 (group) to *n* = 19, 1.6% (highest level of formal education). Finally, we checked for interaction effects between time (representing baseline to endline timepoints) and groups (women with babies, community members, health workers) for each of the factors. All analyses were performed in Stata (version 16.0).

## Results

### Sample characteristics

The final analyses included data from 1202 individuals from two districts in Zambia. Women with babies comprised 32.5%, community members 32.8%, and health workers 34.7% of the total sample. The mean age of participants in the total sample was 38.24 years old (SD = 13.34). Participants reported having lived in the communities where the data was being collected for an average of 26.46 years (SD = 16.12). The majority (86.6%) of participants were married having on average 4.55 (SD = 2.52) living children and very few (5.0%) reported having completed no formal education. Independent variables had missingness that ranged from *n* = 0 (group) to *n* = 19, 1.6% (highest level of formal education). See Table 2 for count, percent, mean and standard deviations of the total sample’s variables separated by intervention and comparison site at baseline and endline.

**Table 2:** Characteristics of ZaMS Intervention and Comparison Sites at Baseline and Endline, *N* = 1202

	ZaMS *n* = 286(51.72%)	Comparison *n* = 267(48.28%)		ZaMS *n* = 366(56.39%)	Comparison 283(43.61%)		Comparing ZaMs Sites from baseline to endline	Comparing Comparison Sites from baseline to endline
	Baseline			Endline				
	n(%)	n(%)	*P*-value	n(%)	n(%)	*P*-value	*P-*value	*P*-value
**Group**			0.35^a^			0.64^a^	0.17^a^	0.13^a^
Women with babies	91(31.82)	98(36.70)		117(31.97)	85(30.04)			
Health workers	98(34.26)	92(34.46)		130(35.52)	96(33.92)			
Community members	97(33.92)	77(28.84)		119(32.51)	102(36.04)			
**Age** (in years), mean (SD)	36.16(12.8)	37.27(13.85)	0.33^b^	39.81(13.55)	39.23(12.82)	0.58^b^	< 0.001	0.09^b^
Sex*			0.29^a^			–		–
Female	209(73.33)	185(69.29)		–	–			
Male	76(26.67)	82(30.71)		–	–			
**Time in the community** (in years), mean (SD)	23(14)	27(18)	0.01^b^	28(16)	27(16)	0.58^b^	< 0.001^b^	0.74^b^
**Marital status**			0.01^a^			0.43	0.77^a^	0.003^a^
Married	241(85.16)	243(92.05)		306(85.96)	231(83.7)			
Unmarried	42(14.84)	21(7.95)		50(14.04)	95(15.03)			
**Number of living children,** mean (SD)	4.27(2.64)	4.47(2.63)	0.37^b^	4.7(2.42)	4.62(2.39)	0.48^b^	0.02^b^	0.49^b^
**Level of education**			0.006^a^			0.72^a^	0.19^a^	0.14^a^
None	14(4.91)	13(5.00)		20(5.54)	12(4.33)			
Some or completed primary	110(38.60)	135(51.92)		163(45.15)	122(44.67)			
Some or completed secondary or tertiary	161(56.49)	112(43.08)		178(49.31)	143(51.62)			

^a^Chi-square test used to compare proportions, ^b^independent t-test used to compare means, *sex only collected at baseline

### Exploratory factor analysis

The factorial structure of the 19-item CM survey was analyzed using various rotations to identify the factor structure most suitable statistically and conceptually for the dataset. It was determined that forcing a factor solution did not improve conceptual clarity and so a final solution was run with principal axis factoring with varimax rotation with items removed that had not loaded highly based on previous iterations. This resulted in four factors with KMO of 0.717 and Bartlett’s showing significance at < 0.01. The four factor structure that emerged in the analysis included constructs of self-efficacy, collective efficacy, power in relationships, and governance (see Table 5 in Appendix for descriptive statistics for each item within domains). This was confirmed by the scree test and the fact that 41% percent of the total variance in the scale was explained by these four factors extracted. Varimax rotation was used, assuming that the four proposed factors are uncorrelated to one another. Each item of the scale loaded cleanly onto the four factors with factor loadings between 0.5–0.8. Cronbach’s alpha for one of the factors was 0.8 (self-efficacy), for two factors was 0.7 (collective efficacy, power in relationships) and for one factor (governance) was 0.6. Conceptually the factors fit together in accordance with the constructs they represent. A table with internal consistency reliability, eigenvalues, communalities, factor loadings, and origin of survey items is present in Table 3.

**Table 3:** Final four factor solution

Factor	Construct	Cronbach’s alpha	Eigenvalue	Communalities (initial, final)	Factor loading	Variable	Original use
1	*Self-efficacy*	0.8	2.75				Romero et al. (2006); Zimmerman & Zahniser(1991)
				(0.37, 0.53)	0.71	I find it very easy to talk in front of groups.	
				(0.39, 0.57)	0.72	I am often a leader in groups.	
				(0.38, 0.50)	0.63	I can usually organize people to get things done.	
2	*Collective efficacy*	0.7	2.03				Romero et al. (2006); Sood (1999)
				(0.28,0.41)	0.64	I believe that a community can talk about the issues that involve them freely among themselves.	
				(0.28,0.47)	0.69	I believe a community can hold group meetings to talk about issues that involve them.	
				(0.16, 0.20)	0.43	I believe a community can work with current community groups to deal with issues that involve them.	
				(0.21,0.28)	0.51	I believe that a community can have a say in changing the conditions of their lives.	
3	*Power in relationships*	0.71	1.66				Romero et al. (2006)
				(0.22,0.31)	0.56	Using a condom every time with my partner would make my partner angry.	
				(0.35,0.57)	0.75	Using a condom every time I have sex would make my partner think I don’t trust them.	
				(0.34,0.51)	0.75	Using a condom every time with my partner would make my partner not trust me.	
4	*Governance*	0.62	1.25				Romero et al. (2006); Israel et al. (1994)
				(0.26,0.40)	0.56	I can influence the decisions that this group makes.	
				(0.21,0.39)	0.62	This group has control over decisions that involve my life.	
				(0.17,0.29)	0.54	This group is successful in achieving its goals.	

* The full survey included questions relevant to four community level CM domains (collective efficacy, governance, perceived control, social acceptance/cohesion) as well as two individual focused domains (power in relationships, self-efficacy) that the parent study compiled from CM interventions focused on sexual and reproductive health. See Table 5 in Appendix for a complete list of domains and questions investigated, with bivariate statistics used to compare individual survey data at baseline and endline between and among sites

### Self-efficacy factor

Bivariate analyses showed statistically significant differences in the mean score of the self-efficacy factor among the intervention sites from baseline to endline (*p* < .01) and among comparison sites from baseline to endline (*p* = .05). The independent variables time, group, age, and level of education were significant in the regression model. The score for the self-efficacy factor increased from baseline to endline by 0.21 (*p* < 0.001). There was no statistically significant difference between the intervention and comparison sites in self-efficacy factor scores. When compared to the women with babies group, the score for the self-efficacy factor was higher for community members by 0.34 (*p* < 0.001). For health workers, the self-efficacy factor score was higher when compared to women with babies by 0.61 (*p* < 0.001). As age increased, self-efficacy factor scores increased for all communities by 0.02 (*p* < 0.001). Compared to those with the highest level of formal education reported, those with no education (b = − 0.85, *p* < 0.001) or having completed some or all of primary school (b = − 0.48, *p* < 0.001) had lower self-efficacy scores.

### Collective efficacy factor

The results of the bivariate analyses exploring differences in mean scores between and among the intervention and comparison sites at baseline and endline showed no statistically significant differences. There were no statistically significant differences between baseline and endline in the collective efficacy factor scores, controlling for all other covariates. The collective efficacy factor regression showed a statistically significant difference between the intervention and comparison sites, with intervention sites showing a decreased score (b = − 0.11, *p* = 0.005). The variable group showed significant differences, with community members (b = 0.21, *p* < 0.001) and health workers (b = 0.21, *p* < 0.001) having higher collective efficacy scores when compared to the women with babies group. Level of formal education was significant in the collective efficacy factor score regression with those having no formal education (b = − 0.26, *p* = 0.008) with lower collective efficacy scores than those participants who reported completing some or all secondary or tertiary education.

### Power in relationships factor

The mean scores between intervention (M = 1.41, SD = 1.22) and comparison (M = 1.78, SD = 1.16) sites showed significant differences at baseline. Similarly, at endline the comparison sites showed statistically significant higher (M = 1.81, SD = 1.17) mean power in relationship scores when compared to the intervention sites (M = 1.58, SD = 1.17). The variables time, site, and number of living children showed statistical significance in the regression model. There was an increase in power in relationships scores from baseline to endline by 0.11 (*p* < 0.001). The intervention communities had decreased power in relationship scores (b = − 0.25, *p* < 0.001), controlling for all other covariates. As the number of living children increased, the power in relationships score decreased by 0.05 (*p* = 0.02).

### Governance factor

There were statistically significant differences between the mean scores of the governance factor among the intervention sites from baseline (M = 2.03, SD = 1.09) to endline (M = 2.53, SD = 0.66). Similarly, there were statistically significant differences in the mean score of the governance factor among the comparison sites from baseline (M = 1.95, SD = 1.17) to endline (M = 2.66, SD = 0.57). The regression analysis showed an increase in the governance factor scores at endline by 0.61 (*p* < 0.001). Health workers had higher governance scores by 0.18 (*p* = 0.03) when compared to women with babies. As the number of living children reported by participants increased, the governance factor decreased by 0.03 (*p* = 0.03). When compared to those who reported having completed some or all secondary or tertiary education, those with no formal education had lower governance scores by 0.37 (*p* = 0.006). For a complete depiction of the results of each factor’s regression, see Table 4.

**Table 4:** Multivariate regression of factors representing CM domains

Covariates	Self-Efficacy		Collective Efficacy		Power in Relationships		Governance	
	β (SE) ***n*** = 1133	B	β (SE) ***n*** = 1119	B	β (SE) ***n*** = 1086	B	β (SE) ***n*** = 1127	B
**Time**								
Baseline	[reference]		[reference]		[reference]		[reference]	
Endline	0.21^a^(.05)	0.10	0.21(.04)	−0.05	0.21(.07)	0.05	0.21^a^(.05)	0.32
**Site**								
Comparison	[reference]		[reference]		[reference]		[reference]	
ZaMS	−0.6(.05)	−0.03	0.21^b^(.04)	−0.08	0.21^a^(.07)	−0.11	0.21(.05)	−0.01
**Group**								
Women with babies	[reference]		[reference]		[reference]		[reference]	
Community members	0.21^a^(.07)	0.15	0.21^a^(.06)	0.15	0.20(.10)	0.05	0.21(.08)	0.07
Health workers	0.21^a^(.08)	0.28	0.21^b^(.06)	0.14	0.21(.11)	−0.08	0.21^b^(.09)	0.09
**Time lived in the community**	0.21(.002)	0.01	0.21(.001)	0.03	0.21(.003)	0.02	0.21^a^(.002)	0.03
**Age**	0.21^a^(.003)	0.22	0.21(.002)	0.05	0.21(.004)	0.06	0.21(.003)	0.06
**Marital status**								
Married	[reference]		[reference]		[reference]		[reference]	
Unmarried	0.21(.08)	0.01	0.21(.06)	0.02	0.21(.11)	−0.05	0.21(.08)	−0.03
**No. living children**	0.21(.003)	0.05	0.21(.01)	0.04	0.21^b^(.02)	−0.10	0.21^b^(.01)	−0.08
**Education**								
None	0.21^a^(.13)	−0.17	0.21^b^(.13)	−0.08	0.21(.19)	−0.002	0.21^b^(.13)	−0.08
Some or completed primary	0.21^a^(.05)	−0.23	0.21(.04)	−0.06	0.21^b^(.08)	0.07	0.21(.06)	0.02
Some or completed secondary or tertiary	[reference]		[reference]		[reference]		[reference]	
	R-square = .32		R-square = .07		R-square = .04		R-square = .23	

*Abbreviations*: *β* standardized beta, *B* unstandardized beta, *No*. Number

^a^Difference is statistically significant at the .001 level

^b^ Difference is statistically significant at the .05 level

## Discussion

Based on these data from two districts in rural Zambia, the CM intervention had an impact at the level of the community among the domains of governance, social cohesion, and the individual domains of self-efficacy and power in relationships. These data reflect CM interventions may function and have impact in different ways for different groups within the same community, with the most marginalized members receiving the least benefit. Women with babies consistently had lower factor scores for the CM domains of collective efficacy, self-efficacy, and governance, mirroring larger patriarchal social and gender norms. These findings reflect the exact presumptions that drive the use of CM in marginalized communities, where it is assumed that women hold less power than others, impacting their ability to take care of their health needs [1]. Manifestations of gender inequity such as these have been reflected in other studies using CM [17], illustrating how women’s low social status influences everything from community priority setting, to household decision making, to the participation of women in CM interventions not directly targeted toward them [18].

Addressing complex problems, such as maternal mortality, requires multifaceted solutions such as CM coupled with MWHs and health facility strengthening, yet reporting and evaluation is a continued challenge. *The Every Woman Every Child Global Strategy for Women’s Children’s and Adolescent’s Health* (2016–2030) has three main calls to action: 1) survive (end preventable deaths), 2) thrive (ensure health and well-being), and 3) transform (expand enabling environments) building on the Sustainable Development Goals agenda [19] .There is a known lack of research around the areas of community participation, social accountability, and those that specifically address gender inequities [19]. Directly targeting women or other marginalized groups or including groups who hold power in communities is a choice that CM researchers must make. Results may appear to emerge more quickly relating to health outcomes when more powerful groups are involved in CM interventions, yet it is necessary to question whether this strategy will subvert harmful social and gender norms in quite the same way, if at all.

Simultaneous strengthening of health systems alongside CM interventions improves health benefits when compared to CM alone [20]. For example, in rural Malawi, health facility quality improvement (e.g., identification of high risk pregnant patients and blood donors, improvement of emergency obstetric referrals) coupled with CM showed a reduction in neonatal death by 22% (OR = 0.78 m 95%CI 0.60–10.1) compared to CM alone [20]. In addition, CM among marginalized women in India showed that women who participated in the intervention had a positive impact on the quality of care that they received when interfacing with health systems [8]. Innovative creation of space for equitable dialogue between health systems and communities they serve has begun to emerge through strategies that build on the successes of CM interventions, such as the social accountability approach using Cooperative for Assistance and Relief Everywhere’s community scorecard [21]. Amplifying these kinds of innovations and further use of CM holds power and potential to increase the delivery of high quality care while mitigating health inequities directly linked to oppressive structures. Future research should continue to explore the impact of simultaneous health systems strengthening and CM interventions in addition to the impact on quality of care. These kinds of dual interventions should be designed using a gender transformative lens to amplify the potential for disrupting broader scale harmful social and gender norms [8].

The health and social implications of widespread implementation of CM approaches have great potential to address the Every Woman Every Child Global Strategy’s mission, yet supportive policy and political will are necessary to unlock these achievements. Policymakers can create space for these interventions by supporting and listening to existing community groups in their jurisdictions with the help of social scientists and health services researchers analyzing which groups are active, meeting on a regular basis, and how they are functioning [22]. Exploring how these groups could be bolstered through use of CM and the community-action cycle through mixed methods or qualitative research could accelerate development of unions between political action and social evolution [22]. Research funding could support these efforts through increased calls for community-engaged research, including CM, intrinsically geared toward advancing health equity.

### Limitations

As a secondary analysis, this study has limitations. The CM survey was comprised of questions generated for and tested among Western populations, this ethnocentrism may have challenged results even among those domains that remained salient following the factor analysis. The collection of participants biological sex (and no collection of gender) only at baseline prohibited us from completing a thorough gendered analysis. Furthermore, data collection was completed by research staff of both sexes. When possible, the women with babies groups worked with female staff, yet this was not always possible and likely influenced the outcome of results. As a secondary analysis using cross sectional data at two timepoints, this study is unable to provide evidence of causality. Furthermore, convenience sampling may have introduced bias, although sampling from multiple groups may have mitigated this to a degree. Individual level constructs of empowerment should be interpreted with caution as they may reflect more on the researcher’s objective interests than the participant’s subjective interests [23]. Finally, the CM intervention in the parent study did not use the community action cycle as a part of their intervention, a suggested key ingredient in developing conscientization [1, 22]. The absence of the community action cycle may have impeded the intervention’s ability to invoke the development of conscientization, and thus, domains such as perceived control may have been unable to properly measure its target.

## Conclusions

Continuing to build the research base for use of CM is vital to expand the use of this strategy for the improvement of maternal and child survival in LMICs, and should continue to be explored to address other complex health and social problems [1, 22]. Consideration should also be given to testing CM for issues such as the maternal mortality crisis in the United States and chronic diseases including heart disease, diabetes, and addiction. Testing adaptable models in the United States and other high-income countries will be important, including the use of technological platforms to facilitate the convening of meetings for the community action cycle.

## Data Availability

The datasets used and/or analysed during the current study are available from the corresponding author on reasonable request.

## Appendix

Table 5.

**Table 5:** Comparing Individual Survey Data Questions at Baseline and Endline between and among Sites, N = 1202

	ZaMS 286(51.72)	Comparison 267(48.28%)		ZaMS 366(56.39%)	Comparison 283(43.61%)		Comparing ZaMs sites baseline to endline	Comparing Comparison sites baseline to endline
Survey items^(n,%, missing)^	Baseline			Endline				
	n(%)	n(%)	*P*-value	n(%)	n(%)	*P*-value	*P*-value	*P*-value
***Self-efficacy***								
I am often a leader in groups ^(5,0.4)^			0.06			0.80	< 0.001	0.08
Yes	169(59.3)	179(67.04)		273(74.79)	207(73.93)			
No	116(40.7)	88(32.96)		92(25.21)	73(26.07)			
I find it very easy to talk in front of groups ^(5,0.4)^			0.58			0.29	< 0.001	0.09
Yes	215(75.44)	206(77.44)		315(86.3)	234(83.27)			
No	70(24.56)	60(22.56)		50(13.7)	47(16.73)			
I can usually organize people to get things done^(8,0.7)^			0.02			0.20	< 0.001	0.21
Yes	208(72.98)	214(81.37)		324(88.77)	240(85.41)			
No	77(27.02)	49(18.63)		41(11.23)	41(14.59)			
***Collective efficacy***								
I believe that a community can talk about the issues that involve them freely among themselves^(10, 0.8)^			0.07			0.03	0.04	0.10
Yes	262(92.25)	254(95.85)		317(87.33)	259(92.5)			
No	22(7.75)	11(4.15)		46(12.67)	21(7.5)			
I believe a community can hold group meetings to talk about issues that involve them^(8, 0.7)^			0.63			0.30	0.01	0.06
Yes	271(95.42)	256(96.24)		328(90.11)	259(92.5)			
No	13(4.58)	10(3.76)		36(9.89)	21(7.5)			
I believe a community can work with current community groups to deal with issues that involve them^(9, 0.7)^			0.22			0.19	0.11	0.14
Yes	264(92.63)	254(95.13)		344(95.56)	274(97.51)			
No	21(7.37)	34(6.16)		16(4.44)	7(2.49)			
I believe that a community can have a say in changing the conditions of their lives^(16, 1.3)^			0.03			0.09	0.10	0.36
Yes	261(92.23)	253(96.56)		345(95.3)	273(97.85)			
No	22(7.77)	9(3.44)		17(4.7)	6(2.15)			
***Power in relationships***								
Using a condom every time I have sex with my partner would make my partner angry^(44,3.7)^			0.03			0.44	0.12	0.98
Yes	124(45.59)	137(54.8)		187(51.8)	151(54.91)			
No	148(54.41)	113(45.2)		174(48.2)	124(45.09)			
Using a condom every time I have sex with my partner would make my partner not trust me^(25,2.1)^			0.04			0.002	0.56	0.72
Yes	147(54.24)	166(62.88)		189(51.92)	179(64.39)			
No	124(45.76)	98(37.12)		175(48.08)	99(35.61)			
Using a condom every time I have sex would make my partner think I don’t trust them^(43,3.6)^			< 0.001			0.02	0.01	0.84
Yes	116(42.8)	157(62.8)		192(53.04)	171(61.96)			
No	155(57.2)	93(37.2)		170(46.96)	105(38.04)			
***Governance***								
I can influence the decisions that this group makes^(14,1.1)^			0.07			0.07	< 0.001	< 0.001
Yes	179(63.03)	147(55.47)		305(83.79)	245(88.77)			
No	105(39.97)	118(44.53)		59(16.21)	31(11.23)			
This group has control over decisions that involve my life^(10, 0.8)^			0.07			< 0.001	< 0.001	< 0.001
Yes	162(56.84)	171(64.29)		283(77.75)	253(91.34)			
No	123(43.16)	95(35.71)		81(22.25)	24(8.66)			
This group is successful in achieving its goals^(14, 1.0)^			0.03			0.01	< 0.001	0.002
Yes	233(82.33)	199(74.81)		334(91.76)	237(89.08)			
No	50(17.67)	67(25.19)		30(8.24)	70(10.92)			

*P*-values calculated using chi squared

## Abbreviations

CAC: Community Action Cycle
CM: Community Mobilization
MWH: Maternity Waiting Homes
SGML: Saving Mothers Giving Life
ZaMS: Zambian Mothers Shelter
